# Quantitative Validation of a Visual Rating Scale for Defining High-Iron Putamen in Patients With Multiple System Atrophy

**DOI:** 10.3389/fneur.2019.01014

**Published:** 2019-09-20

**Authors:** Myung Jun Lee, Tae-Hyung Kim, Seung Joo Kim, Baik-Kyun Kim, Chi-Woong Mun, Jae-Hyeok Lee

**Affiliations:** ^1^Department of Neurology, Pusan National University Hospital, Pusan National University School of Medicine and Biomedical Research Institute, Busan, South Korea; ^2^Research Institute for Convergence of Biomedical Science and Technology, Pusan National University Yangsan Hospital, Yangsan-si, South Korea; ^3^Department of Neurology, Gyeongsang National University Changwon Hospital, Changwon, South Korea; ^4^Department of Neurology, Chungbuk National University Hospital, Cheongju-si, South Korea; ^5^Department of Biomedical Engineering, Inje University, Gimhae-si, South Korea; ^6^Department of Neurology, Research Institute for Convergence of Biomedical Science and Technology, Pusan National University Yangsan Hospital, Pusan National University School of Medicine, Yangsan-si, South Korea

**Keywords:** visual rating scale, multiple system atrophy, putaminal hypointensity, susceptibility weight imaging, brain MR image

## Abstract

**Objectives:** To validate a visual rating scale reflecting sub-regional patterns of putaminal hypointensity in susceptibility-weighted imaging of patients with multiple system atrophy (MSA).

**Methods:** Using a visual rating scale (from G0 to G3), 2 examiners independently rated putaminal hypointensities of 37 MSA patients and 21 control subjects. To investigate the correlation with the scales, R2^*^ values and the volume of the entire putamen were measured.

**Results:** MSA patients with parkinsonian variant had significantly higher scores than those with cerebellar variant. Visual rating scores in MSA were correlated with R2^*^ values [General estimating equation (GEE), Wald chi-square = 25.89, corrected *p* < 0.001] and volume (Wald chi-square = 75.44, corrected *p* < 0.001). They correlated with UPDRS motor scores. Binary logistic regression analyses revealed that the visual rating scale was a significant predictor for discriminating MSA patients from controls [multivariate model adjusted for age and sex, odds ratio 52.722 (corrected *p* = 0.009)]. Pairwise comparison between areas under the curve (AUCs) revealed that the visual rating scale demonstrated higher accuracy than R2^*^ values [difference between AUCs; univariate model = 0.247 (corrected *p* < 0.001); multivariate model = 0.186 (corrected *p* = 0.003)]. There were no significant differences in clinical characteristics between the high-iron group, defined as putamen with visual rating scale ≥ G2 and R2^*^ values ≥ third quartile, and the remaining patients.

**Conclusion:** The visual rating scale, which reflects quantitative iron content and atrophy of the putamen as well as motor severities, could be useful for the discrimination and evaluation of patients with MSA.

## Introduction

Putaminal hypointensity on magnetic resonance imaging (MRI) reflecting pathological iron deposition is a useful sign in differentiating multiple system atrophy (MSA) from Parkinson's disease (PD) (1–4). In the parkinsonian variant of MSA (MSA-P), iron-related hypointense signals, are unevenly distributed and predominantly observed in the posterior putamen with a posterolateral to anteromedial gradient (5).

Among iron-sensitive sequences, susceptibility-weighted imaging (SWI) appears to be the most sensitive for depicting spatial information regarding iron deposition. Previous studies have demonstrated that sub-regional analysis yields higher diagnostic value than the measurement of total iron in the entire putamen. The posterior and inner putamen was the most valuable subregion in differentiating MSA-P from PD (5, 6). On the other hand, visual grading without considering the pattern of spatial distribution failed to differentiate MSA-P from PD (6, 7).

We previously developed a visual rating scale to assess sub-regional patterns of hypointensity in the putamen using SWI (SWI-PUT) by modifying the scale developed by Lee and Baik (2), Harder et al. (8), Wang et al. (9). This scale was found to be valuable for differentiating MSA-P from PD in daily clinical practice (2). However, no study has attempted to validate the scale using quantitative iron measurement in the entire putamen.

In the current study, we validated a SWI-PUT visual rating scale using quantitative measurement of R2^*^ values. To investigate the clinical correlates of high-iron putamen, we used both the visual rating scale for SWI-PUT and quantitative measurement of R2^*^ values to distinguish MSA patients with high-iron putamen.

## Materials and Methods

### Patients

Thirty-seven patients with probable MSA [24 parkinsonian variant (MSA-P), 13 cerebellar variant (MSA-C)] according to international consensus criteria (10), and 21 control subjects from Pusan National University Yangsan Hospital (Yangsan, South Korea) were included. Subjects with vascular lesions or motion artifacts on MRI were excluded. None of the control subjects had a history of head trauma, stroke, or any neurological or psychiatric illnesses. Individuals with spinocerebellar ataxia (SCA) 1, 2, 3, 6, 7, 17, or dentatorubral-pallidoluysian atrophy (DRPLA) were excluded. Disease severity of MSA patients was assessed using the Hoehn and Yahr (H&Y) stage, the motor part of the Unified Parkinson's Disease Rating Scale (UPDRS-III), and the Unified Multiple System Atrophy Rating Scale part II (UMSARS-II). To investigate associations between visual rating scales and contralateral parkinsonian features, we composed UPDRS hemi-scores (sum of unilateral rest tremor, action tremor, bradykinesia and rigidity in UPDRS-III), rigidity hem-scores (sum of upper and lower extremities scores) and bradykinesia hemi-scores (sum of finger tapping, hand movement, rapid alternating movement, and leg agility). This study was approved by the Institutional Review Board of Pusan National University Yangsan Hospital, in accordance with guidelines of the Helsinki Declaration. Written informed consent was obtained from all subjects.

### MRI Acquisition and Analysis

All subjects underwent brain MRI using a 3 Tesla scanner (Verio, Siemens, Erlangen, Germany). The axial scans were set parallel to the intercommissural line. The following sequences were acquired: T1-weighted magnetization prepared rapid acquisition gradient recalled echo (MPRAGE) pulse sequence [repetition time (TR)/echo time (TE)/inversion time (TI), 1900/2.2/900 ms; flip angle (FA), 9°; isotropic voxel size, 1 mm^3^; and acquisition time, 5 min 28 s]; three-dimensional (3D) multi-echo FLASH (fast low angle shot) pulse sequence to acquire R2^*^ images (TR, 24 ms; TE, 2.26/4.91/7.56/10.21/12.86/15.51/18.1/20.81 ms; FA, 6°; and 1 mm^3^ isotropic voxel size); SWI using flow-compensated 3D GRE sequences (TR, 28 ms; TE, 20 ms; FA, 20°; matrix size, 320 × 240; and slice thickness, 2 mm).

R2^*^ maps were calculated using the regression of log signals of the eight multi-echo volumes using customized MATLAB tools (MathWorks Inc., Natick, MA, USA). R2^*^ maps were linearly registered to their T1-weighted images using FLIRT (FMRIB's linear image registration tool). Automatic segmentation of the putamen was performed based on T1-weighted images using FreeSurfer version 5.3 (http://surfer.nmr.mgh.harvard.edu/).

**Figure 1:**
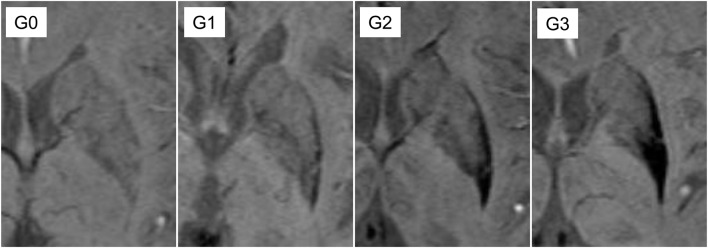
Definition of putaminal visual rating scales. G0: similar hypointensity relative to cerebrospinal fluid (CSF) on the lateral border of putamen; G1: linear hypointensity, darker than CSF or similar to veins, on the lateral margin of putamen; G2: hypointensity with on the posterior putamen with lateral to medial gradient; G3: marked hypointensity on the most posterior half of the putamen and globus pallidus.

The signal intensity and distribution was assessed and scored, as shown in Figure 1 (2) and Supplementary Figure 1. For further clarification, the two consecutive axial slices with the largest area of the putamen containing the anterior commissure, the septum pellucidum, and the pulvinar of thalamus was included. If the grades of the two slices differed, the higher score was selected. In the absence of the lateral-to-medial gradient, linear hypointensity along the lateral border relative to background signal intensity was scored G1.

The visual rating scale for SWI-PUT (2) was evaluated separately by two raters (L.J.H and L.M.J). The inter-rater reliability for the visual rating scale was good (Cohen's kappa = 0.782, approximate SE = 0.046, approximate significance < 0.001). In cases of discrepancy between grades assigned by the two raters, the final grade used in analysis was decided by consensus between the two raters.

### Statistical Analysis

Comparison of continuous variables demonstrating Gaussian distribution was performed using the independent *t*-test. The Mann–Whitney *U*-test was used for variables with non-Gaussian distribution. Categorical variables were compared using the chi-square test. Because putaminal parameters were measured repeatedly within each subject, we employed general estimating equation (GEE) to investigate associations between putaminal rating scales, volume and R2^*^ values. Spearman's correlation test was used to test associations between UPDRS subscores and contralateral visual rating scales.

Binary logistic regression analyses were performed to test whether MRI results may distinguish MSA patients from control subjects. We chose putamen showing worse values (higher visual rating scales or R2^*^ values, and lower volume) as the predictors of logistic regression analyses. In addition, the area under the receiver operating characteristic curves (AUCs) obtained from each binary logistic regression model were compared using a pairwise comparison. Statistical significance was defined as *p* < 0.05. Corrections for multiple testing were performed using the Bonferroni procedure. Statistical analyses were performed using SPSS version 18.0 (IBM Corporation, Armonk, NY, USA) and MedCalc version 18.6 (MedCalc Software, Ostend, Belgium).

## Results

Demographic information and imaging characteristics of the enrolled subjects are summarized in Table 1. MSA-P subjects were predominantly female (male:female = 7:17) compared with those of control subjects, however the difference did not reach statistical significance (12:9; Pearson's chi = 3.593, uncorrected *p* = 0.075). The UPDRS and UMSARS were significantly higher in patients with MSA-P than those with MSA-C. Compared with control subjects, MSA patients had higher visual rating scores regardless of right and left sides (Table 1). When visual rating scores were divided into low (G0 or G1) and high (G2 or G3) grade, 22 MSA-P subjects had either unilateral or bilateral high grade (*n* = 12, unilateral; *n* = 10, bilateral), whereas 2 MSA-C subjects had high grade putamens in the unilateral side. Frequencies of higher or lower visual grade showed significant differences between MSA-P and C groups, regardless of image sides (Table 1). However, there were no significant differences in low and high visual grades in both sides between MSA-C and control subjects (Table 1).

**Table 1:** Demographics, clinical, and imaging characteristics of MSA and control subjects.

	**MSA-total**	**MSA-P**	**MSA-C**	**Controls**	**MSA-total** **vs.** **controls (*p*)**	**MSA-P** **vs.** **MSA-C (*p*)**	**MSA-P** **vs.** **controls (*p*)**	**MSA-C** **vs.** **controls (*p*)**
Age^a^	58.70 ± 6.85	59.00 ± 7.19	58.15 ± 6.27	60.48 ± 6.42	0.341	0.727	0.479	0.316
Gender (M:F)^b^	12:25	7:17	5:8	12:9	0.097	0.716	0.075	0.481
Disease duration (months)^a^	26.57 ± 13.03	27.54 ± 12.76	24.77 ± 13.57	.		0.547		
UPDRS-III^c^	33.62 ± 11.78	37.92 ± 10.95	25.69 ± 8.88	.		0.002^*^		
UMSARS-II^c^	21.84 ± 5.43	23.50 ± 5.32	18.77 ± 4.24	.		0.013^*^		
HandY stage^d^	2.99 ± 0.58	3.06 ± 0.62	2.85 ± 0.46			0.369		
Visual rating (0/1/2/3) ^b^								
Right side	7/13/12/5	1/7/11/5	6/6/1/0	20/1/0/0	< 0.001^*^	0.001^*^	< 0.001^*^	0.002^*^
Left side	9/11/8/9	3/5/7/9	6/6/1/0	20/1/0/0	< 0.001^*^	0.004^*^	< 0.001^*^	0.002^*^
Visual category^†^ (non-higher/ higher grade) ^b^								
Right side	20/17	8 /16	12/1	21/0	< 0.001^*^	0.001^*^	< 0.001^*^	0.382
Left side	20/17	8 /16	12/1	21/0	< 0.001^*^	0.001^*^	< 0.001^*^	0.382
Putaminal R2^*^^e^								
Right side	28.49 ± 6.24	30.37 ± 6.83	25.01 ± 2.65	25.19 ± 3.10	0.008^*^	< 0.001^*^	0.001^*^	0.903
Left side	25.79 ± 5.08	27.05 ± 5.78	23.48 ± 2.07	24.14 ± 3.26				
Putaminal volume^f^								
Right side	3844.61 ± 928.98	3455.83 ± 729.21	4562.35 ± 842.31	5221.04 ± 609.86	< 0.001^*^	< 0.001^*^	< 0.001^*^	0.004^*^
Left side	4168.55 ± 1055.35	3739.57 ± 932.02	4960.52 ± 793.00	5464.26 ± 678.12				

UPDRS-III = Unified Parkinson's Disease Rating Scale part III; UMSARS-II = Unified Multiple System Atrophy Rating Scale part II; HandY stage = Hoehn and Yahr stage;

† = categorization of visual rating (0 or 1 = non-higher category; 2 or 3 = higher category).

a Independent t-test;

b Chi-square test or Fisher's exact test;

c Analysis of Covariance (ANCOVA) test, covariated with age, gender, and disease duration;

d Mann-Whitney U-test;

e General Estimating Equation (GEE) covariated with age, sex and image side;

f GEE covariated with age, sex, total intracranial volume and image side;

^p^Uncorrected p-values for multiple testing;

* *Statistically significant after Bonferroni correction*.

In the GEE analyses using age, sex, and image side as covariate, MSA-total patients had higher R2^*^ values in the putamen than controls. However, there was no significant difference in R2^*^ values between MSA-C patients and control subjects (Table 1). Regardless of clinical subtype, putaminal volumes in MSA patients were significantly lower than those of control subjects.

Regardless of right and left side, visual rating scores showed positive correlations with contralateral UPDRS hemi-scores in overall MSA subjects (right visual rating scores and left UPDRS hemi-scores, Spearman's rho = 0.538, corrected *p* = 0.003; left visual rating scores and right UPDRS hemi-scores, Spearman's rho = 0.473, corrected *p* = 0.009; Supplementary Table 1), although the association between rigidity hemi-scores in the right and visual rating scores in the left became marginal after Bonferroni correction (Spearman's rho = 0.386, corrected *p* = 0.054; Supplementary Table 1). In addition, putaminal volume showed significant negative correlations with contralateral UPDRS subscores, whereas R2^*^ values did not (Supplementary Table 1).

In GEE analyses covariated with age, sex, image side, and interaction between visual rating scores and image side, visual rating scores were significantly correlated with R2^*^ values in MSA-total and MSA-P groups (MSA-total, Wald chi-square = 25.89, β = 4.419, corrected *p* < 0.001; MSA-P, Wald chi-square = 20.91, β = 5.669, corrected *p* < 0.001). In contrast, there was no significant association between R2^*^ values and visual rating scores in the MSA-C patients (Wald chi-square = 0.012, β = −0.049, uncorrected *p* = 0.914). GEE analyses covariated with age, sex, TIV, image side, and interaction between image side and visual rating scores demonstrated significant associations between visual rating scores and putaminal volume in MSA-total (Wald chi-square = 75.435, β = −550.321, corrected *p* < 0.001) and MSA-P patients (Wald chi-square = 49.616, β = −613.167, corrected *p* < 0.001), whereas no correlation was observed in MSA-C patients (Wald chi-square = 3.621, β = −340.431, corrected *p* = 0.171).

**Figure 2:**
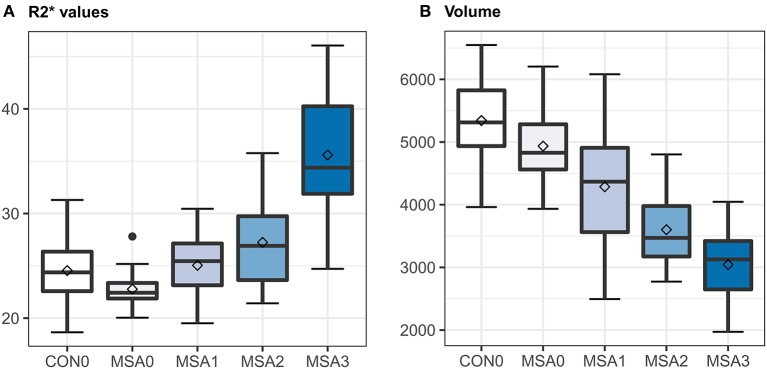
Box and whisker plots illustrating ranges of putaminal R2* values **(A)** and volume **(B)**. CON0 = control putamen with visual rating 0; MSA0, 1, 2, or 3 = MSA putamen with visual rating 0, 1, 2, or 3; horizontal bar in the boxes = median values; squares in the boxes = mean values; error bars = minimal and maximal values.

Notably, there were a considerable overlap of R2^*^ values between each visual rating scale (Figure 2). GEE tests adjusted for age, sex, and image side did not reveal significant differences in putaminal R2^*^ values between G0 and G1, and between G1 and G2. In contrast, putaminal volume showed significant differences between visual rating scales (Supplementary Table 2). In binary logistic regression analyses for discriminating MSA and control subjects, visual rating scores, as well as R2^*^ values and volumes, were a predictor of MSA patients, regardless of whether covariates included in the logistic regression models. However, statistical significance of R2^*^ values did not remain after Bonferroni correction for multiple testing (Table 2). The AUCs of visual rating scores were significantly larger than those of R2^*^ values, whereas putaminal volume had comparable AUCs with the visual rating scale (Table 2).

**Table 2:** Comparisons of binary logistic regression models distinguishing MSA and control subjects.

**Model**	**Predictor**	**β (SE)**	**Odds ratio**	**AUC**	**Sensitivity/** **Specificity**	***p***
Univariate	Visual rating	3.302 (1.070)	27.158	0.911	0.838/0.952	0.002^*^
Univariate	R2*	0.160 (0.070)	1.174	0.664	0.757/0.238	0.022
Univariate	Volume	−0.002 (0.001)	0.998	0.894	0.838/0.714	<0.001^*^
Multivariate^†^	Visual rating	3.965 (1.337)	52.722	0.954	0.865/0.905	0.003^*^
Multivariate^†^	R2*	0.224 (0.094)	1.251	0.768	0.811/0.476	0.018
Multivariate^‡^	Volume	−0.003 (0.001)	0.997	0.925	0.865/0.810	<0.001^*^
**Model**	**Pairwise comparison of AUCs (i vs. j)**	**Differences between AUCs (i – j)**	**SE**	**z**	**95% CI**	***P***
Univariate	Visual rating vs. R2*	0.247	0.064	3.88	0.122 ~ 0.371	<0.001^*^
Univariate	Visual rating vs. volume	0.017	0.041	0.391	−0.065 ~ 0.097	0.696
Univariate	R2* vs. volume	−0.230	0.073	3.162	0.088 ~ 0.373	0.002^*^
Multivariate^†^	Visual rating vs. R2*	0.186	0.057	3.292	0.075 ~ 0.297	0.001^*^
Multivariate^†^	Visual rating vs. volume	0.029	0.030	0.953	−0.030 ~ 0.089	0.341
Multivariate^‡^	R2* vs. volume	−0.157	0.062	2.547	0.036 ~ 0.278	0.011^*^

^β^statistics of binary logistic regression analyses;

^SE^Standard error;

^AUC^area under curve;

^z^z-statistics of pairwise comparison between AUCs;

^CI^Confidence interval;

† covariated with age and gender;

‡ covariated with age, gender and total intracranial volume;

^p^Uncorrected p-values for multiple testing;

* *Statistically significant after Bonferroni correction*.

Finally, we sorted out MSA patients with high-iron putamen, defined as visual rating scale ≥ grade 2, and R2^*^ values ≥ third quartile. The high-iron group exhibited significantly smaller putamen volumes (3492.63 ± 718.16) than those of the remaining patients (4398.16 ± 1014.82; GEE using age, sex, total intracranial volume and image side as covariate, Wald chi-square = 16.389, β = −738.590, corrected *p* < 0.001). However, there were no significant difference in age, sex ratio, disease duration, UMSARS, UPDRS, or H&Y stage between the high-iron group and the remaining patients (Supplementary Table 3).

## Discussion

In this study, the SWI-PUT visual rating scale was validated using quantitative MRI data. This simple method reliably reflected quantitative iron content in the entire putamen and the severity of parkinsonian motor deficits in the contralateral body part. Moreover, the different scores in the rating scale corresponded to different quantitative degrees of atrophy. The SWI-PUT visual rating scale enabled us to easily identify putaminal degeneration specific to MSA.

However, we found substantial mismatch between visual rating scores and quantitative R2^*^ values. There were a wide range of R2^*^ values in the same SWI-PUT grade. Even at high grades, R2^*^ values overlapped with the range of control subjects. These findings can be attributed to the focal and uneven deposition of iron (2, 11, 12). In the opposite case, significant signal hypointensity throughout the putamen might be also found in the absence of the specific visual pattern. In the present study, SWI-PUT grade demonstrated better accuracy in discriminating MSA and control subjects than that by R2^*^ values (Table 2). As mentioned above, consideration of distributional pattern of putaminal hypointensity would be helpful for identifying and classifying MSA patients.

High-iron putamen, defined using two complementary methods, indicate the MSA-P subtype (13, 14). It has been consistently reported that iron-related degeneration in the putamen is more specific to MSA-P (1, 3). In the present study, only volume atrophy was associated with high-iron deposition in the putamen. Similarly, iron accumulation in the putamen increased in parallel with the extent of atrophy in a previous longitudinal study (3). Other clinical variables, including disease duration, were not correlated with high-iron putamen. Due to the unknown period of asymptomatic disease evolution (15), the actual duration of disease may be better reflected by morphological markers of tissue destruction than by symptom duration.

In the present study, putaminal rating scales, and volume showed significant correlations with severities of contralateral parkinsonian features. Previous postmortem studies have demonstrated that parkinsonian motor deficits result from selective neuronal loss and gliosis predominantly affecting striatum and substantia nigra (16, 17). Similarly, neuroimaging studies using diffusion tensor imaging have revealed associations between putaminal mean diffusivity and clinical rating scales such as UPDRS and UMSARS (18–20). Our results suggest that visual rating scales may be useful for monitoring disease progression.

We acknowledge the limitations of the present study. First, we included a relatively small sample size of MSA patients and control subjects. Moreover, the control subjects were not age- and sex- matched with the MSA patients. Second, in the present study, only two raters were participated to measure the visual rating scales. Our results require a cross-validation of further studies with larger sample size. Finally, we did not enroll patients with degenerative parkinsonism aside from MSA. At this time, we are unable to conclude whether our SWI-PUT scales would distinguish MSA from other types of degenerative parkinsonism. However, a previous MRI study using a visual analog scale suggested that putaminal hypointensity and atrophy are also useful for discriminating MSA from PD or progressive supranuclear palsy (21).

In conclusion, a simple SWI-PUT visual rating scale can reflect quantitative iron content and atrophy in the entire putamen. We believe that this scale will be useful for discrimination and evaluation of MSA patients. Recently, iron chelation has been proposed to modify the disease course of MSA (21, 22) and, as such, high-iron putamen may be a potential therapeutic target. Our complementary analysis of pattern recognition and quantitative measurement may be useful for patient selection in this regard.

## Data Availability Statement

All datasets generated for this study are included in the manuscript/Supplementary Files.

## Ethics Statement

The studies involving human participants were reviewed and approved by Institutional Review Boards of Pusan National University Yangsan Hospital. The patients/participants provided their written informed consent to participate in this study.

## Author Contributions

ML: statistical analyses and writing. T-HK: data collection and statistical analyses. SK and B-KK: data collection and revision of manuscript. C-WM: data collection and statistical anlayses. J-HL: study design and writing.

### Conflict of Interest

The authors declare that the research was conducted in the absence of any commercial or financial relationships that could be construed as a potential conflict of interest.
