# Supplementary material for: Quantitative Validation of a Visual Rating Scale for Defining High-Iron Putamen in Patients With Multiple System Atrophy
Source: Front Neurol. 2019 Sep 20;10:1014. doi: 10.3389/fneur.2019.01014 (PMC6763953; doi:10.3389/fneur.2019.01014)
Supplement: Supplementary file 1 [file Data_Sheet_1.PDF]

Supplementary Figure 1.

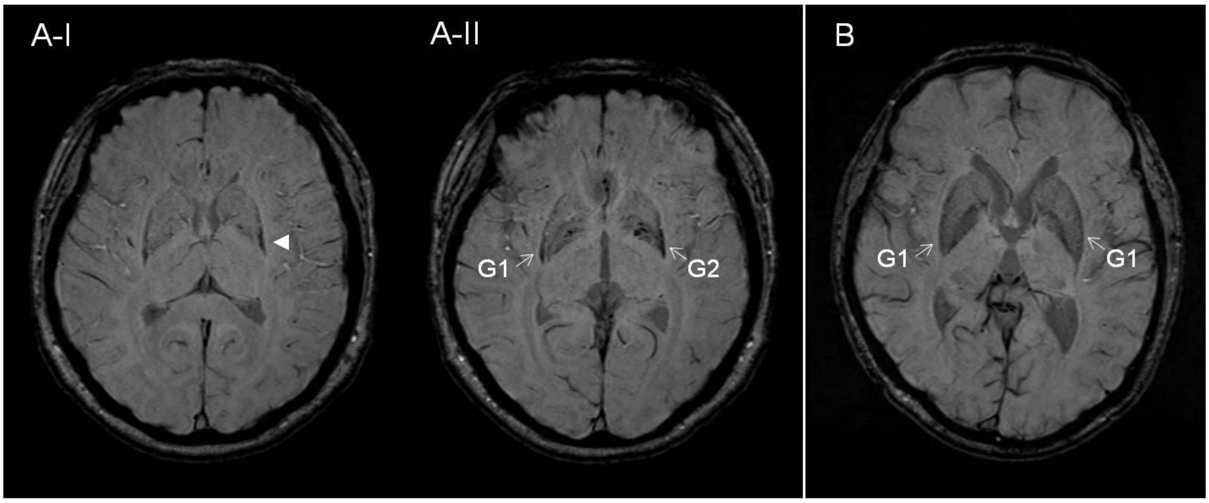

Two consecutive axial slices (A-I, A-II) with the largest area of the putamen containing the anterior commisure, the septum pellucidum, and the pulvinar of thalamus. When accompanied by the straightening of posterolateral margin reflecting volume shrinkage (A-I, arrowhead), we assessed the distribution of hypointensity in the lower axial slice (A-II). Posterolateral signal hypointensity with medial extension was scored as G2. In case of evenly distributed hypointensity throughout the putamen (B), linear hypointensity along the lateral border relative to background signal intensity was scored as G1 because of the absence of a lateral-to-medial gradient. G: grade.

Supplementary Table 1. Correlation matrix between UPDRS subscores and putaminal parameters.

	UPDRS hemi-scores	Rigidity hemi-scores	Bradykinesia hemi-scores
Between right putaminal parameters and left UPDRS subscores			
**Visual rating scores**	0.538 (0.001)*	0.515 (0.001)*	0.483 (0.002)*
**R2* values**	0.200 (0.235)	0.152 (0.370)	0.283 (0.089)
**Volume**	-0.538 (0.001)*	-0.504 (0.001)*	-0.537 (0.001)*
Between left putaminal parameters and right UPDRS subscores			
**Visual rating scores**	0.473 (0.003)*	0.386 (0.018)	0.407 (0.012)*
**R2* values**	0.067 (0.692)	0.005 (0.974)	0.061 (0.721)
**Volume**	-0.529 (0.001)*	-0.411 (0.011)*	-0.526 (0.001)*

Spearman's rho (*p*-values); UPDRS = Unified Parkinson's Disease Rating Scale part III; *p* = uncorrected for multiple testing; * = statistically significant after Bonferroni correction.

Supplementary Table 2. Comparisons of putaminal R2* and volume between visual rating scores in MSA patients

	Putaminal R2*		Putaminal volume	
**Visual rating**	**Mean**	**SD**	**Mean**	**SD**
0	22.77	1.83	4935.34	645.31
1	25.04	2.91	4286.02	1003.41
2	27.24	4.31	3601.79	606.48
3	35.59	5.90	3044.37	607.56
***post hoc* (*i vs. j*)**	**Difference between EMM (SE)[a]**	***p***	**Difference between EMM (SE)[b]**	***p***
0 *vs.* 1	-1.139 (0.891)	0.201	594.085 (208.670)	0.004*
0 *vs.* 2	-2.518 (0.914)	0.006*	949.800 (177.668)	< 0.001*
0 *vs.* 3	-11.128 (1.319)	< 0.001*	1725.583 (185.183)	< 0.001*
1 *vs.* 2	-1.379 (0.827)	0.095	355.715 (117.719)	0.003*
1 *vs.* 3	-9.989 (1.547)	< 0.001*	1131.498 (127.749)	< 0.001*
2 *vs.* 3	-8.610 (1.287)	< 0.001*	775.783 (107.608)	< 0.001*

EMM = estimated marginal mean values from general estimating equation (GEE) model (i - j); SE = standard error; a = GEE model covariated with age, sex and image side; b = GEE model covariated with age, sex, total intracranial volume and image side; *p* = uncorrected p-values for multiple testing; * = statistically significant after Bonferroni correction.

Supplementary Table 3. Comparison of clinical and imaging parameters between non-higher and higher iron groups

	**Non-higher iron group**	**Higher iron group**	***p***
**Age**[a]	57.86 ± 7.70	59.81 ± 5.72	0.280
**Gender** (M:F)[b]	9:12	3:13	0.166
**Disease duration** (months)[a]	23.67 ± 11.98	30.38 ± 13.94	0.130
**UMSARS-II**[a]	21.05 ± 5.33	22.88 ± 5.64	0.294
**UPDRS-III**[a]	31.76 ± 11.58	36.06 ± 12.15	0.387
**H&Y stage**[a]	2.93 ± 0.53	3.06 ± 0.66	0.457
**Putaminal R2* values**[c]	23.70 ± 2.43	31.66 ± 5.87	< 0.001[*]
**Putaminal volume**[d]	4398.16 ± 1014.82	3492.63 ± 718.16	< 0.001[*]

UMSARS-II = Unified Multiple System Atrophy Rating Scale, part II; UPDRS-III = Unified Parkinson's Disease Rating Scale, part III; H&Y stage = Hoehn and Yahr stage; a = Mann-Whitney U test; b = Fisher's exact test; c = general estimating equation (GEE) test, covariated with age, sex and image side; d = GEE test covariated with age, sex, total intracranial volume and image side; *p* = uncorrected *p*-values for multiple testing; * = statistically significant after Bonferroni correction.
